# *cis*,*trans*-Dicarbonyl­dichlorido[2-(2-pyrid­yl)-1,8-naphthyridine-κ*N* ^1^,*N* ^2^]ruthenium(II)

**DOI:** 10.1107/S1600536808003188

**Published:** 2008-02-06

**Authors:** Dai Oyama, Takashi Hamada

**Affiliations:** a Department of Industrial Systems Engineering, Cluster of Science and Technology, Fukushima University, 1 Kanayagawa, Fukushima 960-1296, Japan; b Department of Science Education, Faculty of Education, Fukushima University, 1 Kanayagawa, Fukushima 960-1296, Japan

## Abstract

The asymmetric unit of the title compound, [RuCl_2_(C_13_H_9_N_3_)(CO)_2_], consists of four crystallographically independent Ru^II^ complexes. Each Ru^II^ atom is in a distorted octa­hedral environment coordinated by two carbonyl ligands, two Cl atoms and a chelating 2-(2-pyrid­yl)-1,8-naphthyridine (pynp) ligand. The carbonyl ligands are *cis* to each other, while the Cl atoms are *trans*. Relatively short inter­atomic distances (2.60–2.67 Å) between the uncoordinated N atom of pynp and the C atom of the carbonyl imply a donor–acceptor inter­action between the pynp and carbonyl ligands.

## Related literature

For related synthetic details, see: Anderson *et al.* (1995 ▶); Campos-Fernandez *et al.* (2002 ▶). For related structures, see: Haukka *et al.* (1995 ▶); Tomon *et al.* (2005 ▶). For related literature on the redox behavior of ruthenium polypyridyl complexes with a 1,8-naphthyridine ligand, see: Nakajima & Tanaka (1995 ▶); Mizukawa *et al.* (1999 ▶); Tomon *et al.* (2005 ▶). For general background on the photochemical reduction of CO_2_, see: Lehn & Ziesel (1990 ▶).
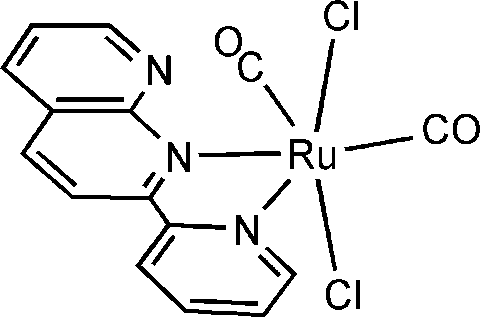


## Experimental

### 

#### Crystal data

[RuCl_2_(C_13_H_9_N_3_)(CO)_2_]
*M* *_r_* = 435.23
Monoclinic, 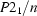

*a* = 16.6297 (14) Å
*b* = 21.6048 (14) Å
*c* = 19.0585 (16) Å
β = 114.082 (3)°
*V* = 6251.4 (9) Å^3^
*Z* = 16
Mo *K*α radiation
μ = 1.36 mm^−1^
*T* = 173 (1) K
0.20 × 0.20 × 0.20 mm

#### Data collection

Rigaku/MSC Mercury CCD diffractometer
Absorption correction: none
49009 measured reflections
14039 independent reflections
12059 reflections with *F* ^2^ > 2σ(*F* ^2^)
*R*_int_ = 0.063

#### Refinement

*R*[*F* ^2^ > 2σ(*F* ^2^)] = 0.078
*wR*(*F* ^2^) = 0.152
*S* = 1.50
14038 reflections
829 parameters
H-atom parameters constrained
Δρ_max_ = 1.43 e Å^−3^
Δρ_min_ = −1.40 e Å^−3^

### 

Data collection: *CrystalClear* (Rigaku/MSC, 2001 ▶); cell refinement: *CrystalClear*; data reduction: *TEXSAN* (Rigaku/MSC, 2000 ▶); program(s) used to solve structure: *DIRDIF94* (Beurskens *et al.*, 1994 ▶); program(s) used to refine structure: *TEXSAN*; molecular graphics: *ORTEP-3 for Windows* (Farrugia, 1997 ▶); software used to prepare material for publication: *CrystalStructure* (Rigaku/MSC, 2004 ▶).

## Supplementary Material

Crystal structure: contains datablocks global, I. DOI: 10.1107/S1600536808003188/is2275sup1.cif

Structure factors: contains datablocks I. DOI: 10.1107/S1600536808003188/is2275Isup2.hkl

Additional supplementary materials:  crystallographic information; 3D view; checkCIF report

## Figures and Tables

**Table 1:** Selected bond lengths (Å)

Ru1—Cl1	2.3933 (12)
Ru1—Cl2	2.3968 (13)
Ru1—N1	2.124 (5)
Ru1—N2	2.148 (4)
Ru1—C1	1.901 (7)
Ru1—C2	1.880 (6)
Ru2—Cl3	2.3883 (13)
Ru2—Cl4	2.3992 (13)
Ru2—N4	2.121 (5)
Ru2—N5	2.129 (4)
Ru2—C16	1.903 (7)
Ru2—C17	1.865 (6)
Ru3—Cl5	2.3996 (13)
Ru3—Cl6	2.3955 (12)
Ru3—N7	2.113 (5)
Ru3—N8	2.160 (4)
Ru3—C31	1.900 (7)
Ru3—C32	1.851 (6)
Ru4—Cl7	2.3955 (13)
Ru4—Cl8	2.3953 (13)
Ru4—N10	2.124 (5)
Ru4—N11	2.128 (4)
Ru4—C46	1.884 (7)
Ru4—C47	1.863 (6)

**Fig. 1.**
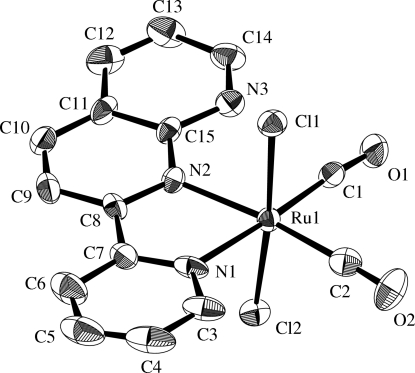
The molecular structure of the title compound, with atom labels and 50% probability displacement ellipsoids for non-H atoms. H atoms have been omitted for clarity.

## supplementary crystallographic information

### Comment

[Ru(bpy)(CO)_2_Cl_2_] (bpy = 2,2'-bipyridine) is known as an excellent catalyst for the photochemical reduction of CO_2_ into formate (Lehn & Ziesel, 1990). On the other hand, some interesting redox properties of ruthenium polypyridyl complexes with a 1,8-naphthyridine (napy) as a redox active ligand have been reported so far (Nakajima & Tanaka, 1995; Mizukawa *et al.*, 1999; Tomon *et al.*, 2005). In the present work, [Ru(pynp)(CO)_2_Cl_2_] [pynp = 2-(2-pyridyl)-1,8-naphthyridine] was newly prepared. Pynp (Campos-Fernandez *et al.*, 2002) is a ligand which has the combined properties of 2,2'-bipyridine and 1,8-naphthyridine.

The crystal structure of the title compound contains four [Ru(pynp)(CO)_2_Cl_2_] complexes of crystallization in the asymmetric unit. The ruthenium(II) complex displays a *cis* orientation of the carbonyl ligands and a *trans* orientation of the chloro ligands (Fig. 1). Two Ru—C—O bond angles of the complex [173.4 (5)–177.7 (7)°] are nearly linear, and the C—O [1.126 (9)–1.150 (7) Å], Ru—Cl [2.3883 (13)–2.3996 (13) Å], Ru—N [2.113 (5)–2.160 (4) Å] distances are in the expected ranges (Table 1). On the other hand, the Ru—C bond distances [1.851 (6)–1.903 (7) Å] are longer than those of [Ru(bpy)(CO)_2_Cl_2_] [1.817 (8), 1.835 (17) Å; Haukka *et al.*, 1995]. The naphthyridine moiety of the pynp ligand is directed toward the adjacent terminal carbonyl ligand. The relatively short interatomic distances between the non-bonded nitrogen atom of pynp and the carbon atom of one of carbonyls [2.60—2.67 Å] are comparable to that of [Ru(bpy)_2_(napy)(CO)]^2+^ [2.765 (7) Å; Tomon *et al.*, 2005], which exhibits intramolecular metallacyclization between the non-bonded nitrogen atom of the napy ligand and the carbonyl carbon atom driven by the napy-based redox reaction.

### Experimental

A methanol solution (10 ml) containing [Ru(CO)_2_Cl_2_]_n_ (31 mg) and pynp (30 mg) was refluxed for 1.5 h. The reaction mixture was then concentrated to 2 ml under reduced pressure. The yellow-green color precipitate was collected by filtration and washed with methanol and diethyl ether, and then dried under vacuum. Single crystals suitable for X-ray diffraction were prepared by the diffusion of diethyl ether into an acetonitrile solution of the complex over a week.

### Refinement

All H atoms were positioned geometrically (C—H = 0.95 Å) and refined as riding, with *U*_iso_(H) = 1.2*U*_eq_(C). Refinement was carried out using reflections with F^2^ > 0.0σ(F^2^).

### Crystal data

**Table:**

[RuCl_2_(C_13_H_9_N_3_)(CO)_2_]	*F*_000_ = 3424.00
*M**_r_* = 435.23	*D*_x_ = 1.850 Mg m^−^^3^
Monoclinic, *P*2_1_/*n*	Mo *K*α radiation λ = 0.71070 Å
Hall symbol: -P 2yn	Cell parameters from 13888 reflections
*a* = 16.6297 (14) Å	θ = 3.1–27.5º
*b* = 21.6048 (14) Å	µ = 1.36 mm^−^^1^
*c* = 19.0585 (16) Å	*T* = 173 (1) K
β = 114.082 (3)º	Prism, yellow–green
*V* = 6251.4 (9) Å^3^	0.20 × 0.20 × 0.20 mm
*Z* = 16	

### Data collection

**Table:**

Rigaku/MSC Mercury CCD diffractometer	12059 reflections with *F*^2^ > 2σ(*F*^2^)
Detector resolution: 14.62 pixels mm^-1^	*R*_int_ = 0.063
ω scans	θ_max_ = 27.5º
Absorption correction: none	*h* = −21→21
49009 measured reflections	*k* = −26→28
14039 independent reflections	*l* = −24→24

### Refinement

**Table:**

Refinement on *F*^2^	H-atom parameters constrained
*R*[*F*^2^ > 2σ(*F*^2^)] = 0.078	*w* = 1/[0.001*F*_o_^2^ + 3σ(*F*_o_^2^) + 0.5]/(4*F*_o_^2^)
*wR*(*F*^2^) = 0.152	(Δ/σ)_max_ = 0.001
*S* = 1.50	Δρ_max_ = 1.43 e Å^−^^3^
14038 reflections	Δρ_min_ = −1.40 e Å^−^^3^
829 parameters	Extinction correction: none

### Special details

**Table:**

Refinement. Refinement using reflections with *F*^2^ > 2.0 σ(*F*^2^). The weighted *R*-factor (*wR*) and goodness of fit (*S*) are based on *F*^2^. *R*-factor (gt) are based on *F*. The threshold expression of *F*^2^ > 2.0 σ(*F*^2^) is used only for calculating *R*-factor (gt).

### Fractional atomic coordinates and isotropic or equivalent isotropic displacement parameters (Å^2^)

**Table:**

	*x*	*y*	*z*	*U*_iso_*/*U*_eq_	
Ru1	0.17626 (3)	0.20501 (2)	0.48235 (2)	0.02146 (12)	
Ru2	0.26702 (3)	0.52889 (2)	0.25696 (2)	0.02210 (12)	
Ru3	0.17269 (3)	0.18945 (2)	−0.02197 (2)	0.02201 (12)	
Ru4	0.24140 (3)	0.52453 (2)	−0.23420 (2)	0.02233 (12)	
Cl1	0.06386 (9)	0.19503 (6)	0.35501 (8)	0.0272 (3)	
Cl2	0.29508 (9)	0.22208 (6)	0.60516 (8)	0.0303 (3)	
Cl3	0.37956 (10)	0.49259 (7)	0.37431 (8)	0.0325 (4)	
Cl4	0.15511 (10)	0.55566 (7)	0.13358 (8)	0.0313 (4)	
Cl5	0.06215 (9)	0.17790 (7)	−0.15037 (8)	0.0315 (4)	
Cl6	0.29082 (9)	0.20463 (6)	0.10162 (8)	0.0272 (3)	
Cl7	0.13015 (10)	0.55219 (6)	−0.35724 (8)	0.0315 (3)	
Cl8	0.34896 (9)	0.48759 (7)	−0.11404 (8)	0.0309 (3)	
O1	0.0528 (3)	0.1442 (2)	0.5419 (2)	0.0419 (14)	
O2	0.2435 (3)	0.07537 (19)	0.4855 (2)	0.0370 (13)	
O3	0.1594 (3)	0.5568 (2)	0.3487 (2)	0.0390 (13)	
O4	0.3353 (3)	0.6589 (2)	0.2864 (2)	0.0458 (15)	
O5	0.0451 (3)	0.1371 (2)	0.0398 (2)	0.0490 (15)	
O6	0.2341 (3)	0.0586 (2)	−0.0194 (2)	0.0459 (14)	
O7	0.1349 (3)	0.5783 (2)	−0.1535 (2)	0.0466 (15)	
O8	0.3278 (3)	0.6493 (2)	−0.2133 (2)	0.0496 (15)	
N1	0.2545 (2)	0.2500 (2)	0.4331 (2)	0.0235 (12)	
N2	0.1470 (2)	0.3014 (2)	0.4864 (2)	0.0217 (12)	
N3	0.0393 (3)	0.2824 (2)	0.5304 (2)	0.0273 (13)	
N4	0.3478 (2)	0.5031 (2)	0.1998 (2)	0.0242 (12)	
N5	0.2342 (3)	0.4342 (2)	0.2289 (2)	0.0221 (12)	
N6	0.1209 (3)	0.4358 (2)	0.2691 (2)	0.0270 (13)	
N7	0.2548 (2)	0.2286 (2)	−0.0705 (2)	0.0229 (12)	
N8	0.1508 (2)	0.2878 (2)	−0.0192 (2)	0.0225 (12)	
N9	0.0415 (3)	0.2756 (2)	0.0245 (2)	0.0338 (15)	
N10	0.3154 (3)	0.4833 (2)	−0.2900 (2)	0.0299 (14)	
N11	0.1944 (3)	0.4317 (2)	−0.2534 (2)	0.0237 (12)	
N12	0.0846 (3)	0.4522 (2)	−0.2121 (2)	0.0300 (14)	
C1	0.0986 (4)	0.1699 (2)	0.5221 (3)	0.0287 (16)	
C2	0.2171 (3)	0.1240 (2)	0.4817 (3)	0.0265 (16)	
C3	0.3024 (3)	0.2213 (2)	0.4009 (3)	0.0275 (16)	
C4	0.3575 (3)	0.2538 (3)	0.3755 (3)	0.0328 (17)	
C5	0.3647 (3)	0.3165 (3)	0.3837 (3)	0.0314 (17)	
C6	0.3145 (3)	0.3469 (2)	0.4158 (3)	0.0286 (16)	
C7	0.2584 (3)	0.3125 (2)	0.4388 (3)	0.0251 (15)	
C8	0.1980 (3)	0.3408 (2)	0.4695 (3)	0.0255 (15)	
C9	0.1934 (4)	0.4047 (2)	0.4771 (3)	0.0298 (16)	
C10	0.1364 (4)	0.4294 (2)	0.5042 (3)	0.0312 (16)	
C11	0.0804 (3)	0.3891 (2)	0.5228 (3)	0.0262 (15)	
C12	0.0190 (4)	0.4090 (2)	0.5517 (3)	0.0319 (16)	
C13	−0.0312 (3)	0.3665 (3)	0.5673 (3)	0.0322 (17)	
C14	−0.0183 (3)	0.3038 (3)	0.5565 (3)	0.0317 (17)	
C15	0.0888 (3)	0.3250 (2)	0.5135 (3)	0.0212 (14)	
C16	0.1963 (4)	0.5453 (2)	0.3120 (3)	0.0285 (16)	
C17	0.3079 (4)	0.6102 (2)	0.2762 (3)	0.0301 (17)	
C18	0.3990 (3)	0.5412 (2)	0.1807 (3)	0.0274 (15)	
C19	0.4506 (3)	0.5218 (3)	0.1436 (3)	0.0340 (17)	
C20	0.4517 (4)	0.4605 (3)	0.1271 (3)	0.0390 (19)	
C21	0.3998 (4)	0.4196 (2)	0.1471 (3)	0.0357 (18)	
C22	0.3472 (3)	0.4423 (2)	0.1825 (3)	0.0272 (15)	
C23	0.2847 (3)	0.4038 (2)	0.2001 (3)	0.0228 (14)	
C24	0.2748 (4)	0.3405 (2)	0.1839 (3)	0.0289 (16)	
C25	0.2118 (4)	0.3078 (2)	0.1962 (3)	0.0316 (17)	
C26	0.1582 (4)	0.3375 (2)	0.2271 (3)	0.0280 (16)	
C27	0.0924 (4)	0.3081 (2)	0.2431 (3)	0.0364 (18)	
C28	0.0434 (4)	0.3433 (3)	0.2707 (3)	0.0383 (19)	
C29	0.0595 (4)	0.4064 (3)	0.2827 (3)	0.0351 (18)	
C30	0.1705 (3)	0.4018 (2)	0.2417 (3)	0.0247 (15)	
C31	0.0923 (4)	0.1593 (2)	0.0177 (3)	0.0315 (17)	
C32	0.2082 (4)	0.1082 (2)	−0.0216 (3)	0.0285 (16)	
C33	0.2991 (4)	0.1963 (3)	−0.1035 (3)	0.0320 (17)	
C34	0.3558 (4)	0.2241 (3)	−0.1298 (3)	0.041 (2)	
C35	0.3681 (4)	0.2869 (3)	−0.1227 (3)	0.042 (2)	
C36	0.3212 (4)	0.3214 (3)	−0.0905 (3)	0.0381 (19)	
C37	0.2633 (3)	0.2914 (2)	−0.0657 (3)	0.0244 (15)	
C38	0.2055 (3)	0.3238 (2)	−0.0365 (3)	0.0254 (15)	
C39	0.2083 (4)	0.3883 (2)	−0.0276 (3)	0.0358 (18)	
C40	0.1516 (4)	0.4168 (2)	−0.0026 (3)	0.043 (2)	
C41	0.0926 (4)	0.3805 (3)	0.0156 (3)	0.0371 (18)	
C42	0.0311 (5)	0.4049 (3)	0.0423 (3)	0.047 (2)	
C43	−0.0223 (4)	0.3649 (4)	0.0575 (3)	0.052 (2)	
C44	−0.0142 (4)	0.3012 (3)	0.0495 (3)	0.045 (2)	
C45	0.0930 (3)	0.3147 (2)	0.0063 (3)	0.0261 (15)	
C46	0.1725 (3)	0.5554 (2)	−0.1843 (3)	0.0277 (16)	
C47	0.2939 (4)	0.6024 (3)	−0.2205 (3)	0.0332 (17)	
C48	0.3730 (4)	0.5126 (3)	−0.3110 (3)	0.041 (2)	
C49	0.4207 (4)	0.4819 (4)	−0.3452 (3)	0.051 (2)	
C50	0.4124 (4)	0.4188 (4)	−0.3541 (4)	0.058 (2)	
C51	0.3538 (4)	0.3882 (3)	−0.3325 (3)	0.049 (2)	
C52	0.3036 (4)	0.4213 (3)	−0.3025 (3)	0.0336 (17)	
C53	0.2367 (3)	0.3931 (2)	−0.2814 (3)	0.0265 (15)	
C54	0.2144 (4)	0.3291 (2)	−0.2920 (3)	0.0367 (18)	
C55	0.1492 (4)	0.3065 (2)	−0.2738 (4)	0.0398 (19)	
C56	0.1030 (4)	0.3463 (2)	−0.2453 (3)	0.0337 (17)	
C57	0.0326 (4)	0.3282 (3)	−0.2259 (4)	0.047 (2)	
C58	−0.0097 (4)	0.3712 (3)	−0.2026 (4)	0.045 (2)	
C59	0.0205 (4)	0.4325 (3)	−0.1949 (3)	0.0374 (19)	
C60	0.1265 (3)	0.4096 (2)	−0.2367 (3)	0.0260 (15)	
H1	0.2985	0.1775	0.3952	0.033*	
H2	0.3903	0.2323	0.3524	0.039*	
H3	0.4034	0.3390	0.3677	0.038*	
H4	0.3182	0.3906	0.4222	0.034*	
H5	0.2302	0.4311	0.4632	0.036*	
H6	0.1341	0.4729	0.5106	0.037*	
H7	0.0129	0.4518	0.5601	0.038*	
H8	−0.0743	0.3789	0.5852	0.039*	
H9	−0.0531	0.2744	0.5687	0.038*	
H10	0.3999	0.5838	0.1933	0.033*	
H11	0.4847	0.5507	0.1298	0.041*	
H12	0.4874	0.4459	0.1024	0.047*	
H13	0.4002	0.3766	0.1367	0.043*	
H14	0.3120	0.3203	0.1643	0.035*	
H15	0.2040	0.2649	0.1839	0.038*	
H16	0.0821	0.2649	0.2349	0.044*	
H17	−0.0018	0.3246	0.2817	0.046*	
H18	0.0241	0.4296	0.3017	0.042*	
H19	0.2909	0.1528	−0.1089	0.038*	
H20	0.3863	0.2000	−0.1528	0.049*	
H21	0.4081	0.3066	−0.1395	0.050*	
H22	0.3286	0.3650	−0.0854	0.046*	
H23	0.2495	0.4121	−0.0390	0.043*	
H24	0.1519	0.4606	0.0024	0.051*	
H25	0.0275	0.4482	0.0494	0.056*	
H26	−0.0656	0.3802	0.0736	0.063*	
H27	−0.0511	0.2745	0.0629	0.054*	
H28	0.3815	0.5558	−0.3021	0.050*	
H29	0.4586	0.5042	−0.3624	0.062*	
H30	0.4469	0.3965	−0.3749	0.070*	
H31	0.3476	0.3446	−0.3381	0.059*	
H32	0.2453	0.3021	−0.3118	0.044*	
H33	0.1348	0.2637	−0.2803	0.048*	
H34	0.0155	0.2860	−0.2292	0.056*	
H35	−0.0588	0.3603	−0.1917	0.054*	
H36	−0.0079	0.4620	−0.1758	0.045*	

### Atomic displacement parameters (Å^2^)

**Table:**

	*U*^11^	*U*^22^	*U*^33^	*U*^12^	*U*^13^	*U*^23^
Ru1	0.0231 (2)	0.0193 (2)	0.0243 (2)	0.00155 (18)	0.0120 (2)	0.00087 (18)
Ru2	0.0253 (2)	0.0177 (2)	0.0249 (2)	0.00077 (18)	0.0119 (2)	−0.00137 (18)
Ru3	0.0232 (2)	0.0202 (2)	0.0247 (2)	0.00118 (18)	0.0120 (2)	0.00049 (19)
Ru4	0.0232 (2)	0.0211 (2)	0.0242 (2)	−0.00135 (18)	0.0112 (2)	−0.00071 (18)
Cl1	0.0265 (7)	0.0283 (7)	0.0276 (7)	−0.0001 (5)	0.0117 (6)	0.0016 (6)
Cl2	0.0304 (8)	0.0308 (7)	0.0272 (7)	0.0050 (6)	0.0094 (6)	−0.0026 (6)
Cl3	0.0310 (8)	0.0315 (7)	0.0306 (8)	−0.0013 (6)	0.0080 (6)	0.0037 (6)
Cl4	0.0304 (8)	0.0338 (8)	0.0299 (8)	0.0044 (6)	0.0125 (6)	0.0054 (6)
Cl5	0.0285 (7)	0.0334 (7)	0.0306 (8)	−0.0013 (6)	0.0099 (6)	0.0015 (6)
Cl6	0.0270 (7)	0.0267 (7)	0.0276 (7)	0.0037 (5)	0.0107 (6)	−0.0029 (5)
Cl7	0.0298 (7)	0.0307 (7)	0.0301 (8)	0.0007 (6)	0.0084 (6)	0.0035 (6)
Cl8	0.0280 (7)	0.0340 (7)	0.0285 (8)	−0.0010 (6)	0.0093 (6)	0.0036 (6)
O1	0.045 (2)	0.035 (2)	0.059 (3)	−0.001 (2)	0.036 (2)	0.013 (2)
O2	0.048 (2)	0.022 (2)	0.043 (2)	0.011 (2)	0.021 (2)	0.0034 (19)
O3	0.050 (2)	0.033 (2)	0.050 (2)	−0.005 (2)	0.036 (2)	−0.012 (2)
O4	0.073 (3)	0.024 (2)	0.058 (3)	−0.013 (2)	0.044 (2)	−0.009 (2)
O5	0.040 (2)	0.057 (3)	0.061 (3)	−0.004 (2)	0.031 (2)	0.023 (2)
O6	0.061 (3)	0.027 (2)	0.042 (2)	0.012 (2)	0.012 (2)	0.001 (2)
O7	0.043 (2)	0.049 (3)	0.055 (3)	−0.004 (2)	0.028 (2)	−0.024 (2)
O8	0.064 (3)	0.034 (2)	0.050 (3)	−0.021 (2)	0.022 (2)	−0.001 (2)
N1	0.020 (2)	0.027 (2)	0.026 (2)	0.000 (2)	0.011 (2)	0.000 (2)
N2	0.021 (2)	0.020 (2)	0.025 (2)	0.0022 (18)	0.010 (2)	−0.0024 (19)
N3	0.028 (2)	0.028 (2)	0.027 (2)	0.003 (2)	0.012 (2)	−0.001 (2)
N4	0.024 (2)	0.023 (2)	0.028 (2)	0.002 (2)	0.013 (2)	−0.001 (2)
N5	0.026 (2)	0.018 (2)	0.021 (2)	0.0011 (19)	0.008 (2)	0.0019 (19)
N6	0.025 (2)	0.027 (2)	0.031 (2)	−0.001 (2)	0.012 (2)	0.000 (2)
N7	0.019 (2)	0.031 (2)	0.017 (2)	0.003 (2)	0.006 (2)	0.000 (2)
N8	0.019 (2)	0.023 (2)	0.023 (2)	0.0040 (19)	0.007 (2)	−0.0005 (19)
N9	0.022 (2)	0.049 (3)	0.032 (2)	0.004 (2)	0.012 (2)	−0.007 (2)
N10	0.017 (2)	0.049 (3)	0.025 (2)	0.007 (2)	0.011 (2)	0.002 (2)
N11	0.027 (2)	0.023 (2)	0.021 (2)	−0.001 (2)	0.009 (2)	−0.0041 (19)
N12	0.030 (2)	0.029 (2)	0.036 (2)	−0.002 (2)	0.019 (2)	0.002 (2)
C1	0.035 (3)	0.027 (3)	0.028 (3)	0.008 (2)	0.018 (2)	0.006 (2)
C2	0.025 (3)	0.031 (3)	0.022 (3)	−0.002 (2)	0.008 (2)	0.004 (2)
C3	0.022 (3)	0.032 (3)	0.031 (3)	0.003 (2)	0.014 (2)	0.001 (2)
C4	0.024 (3)	0.048 (4)	0.030 (3)	0.008 (2)	0.015 (2)	0.002 (2)
C5	0.017 (2)	0.050 (3)	0.025 (3)	−0.004 (2)	0.007 (2)	0.009 (2)
C6	0.025 (3)	0.034 (3)	0.024 (3)	−0.001 (2)	0.007 (2)	0.003 (2)
C7	0.023 (3)	0.025 (2)	0.029 (3)	0.000 (2)	0.012 (2)	0.002 (2)
C8	0.023 (3)	0.028 (3)	0.025 (3)	−0.003 (2)	0.009 (2)	0.002 (2)
C9	0.033 (3)	0.023 (3)	0.033 (3)	−0.003 (2)	0.014 (2)	0.001 (2)
C10	0.038 (3)	0.020 (2)	0.033 (3)	0.003 (2)	0.013 (3)	−0.003 (2)
C11	0.020 (2)	0.029 (3)	0.025 (3)	0.004 (2)	0.004 (2)	0.000 (2)
C12	0.033 (3)	0.030 (3)	0.027 (3)	0.013 (2)	0.006 (2)	−0.002 (2)
C13	0.026 (3)	0.041 (3)	0.032 (3)	0.004 (2)	0.013 (2)	−0.005 (2)
C14	0.024 (3)	0.042 (3)	0.032 (3)	−0.003 (2)	0.016 (2)	−0.004 (2)
C15	0.017 (2)	0.024 (2)	0.020 (2)	0.004 (2)	0.005 (2)	0.000 (2)
C16	0.035 (3)	0.020 (2)	0.025 (3)	−0.004 (2)	0.007 (2)	−0.003 (2)
C17	0.037 (3)	0.026 (3)	0.036 (3)	0.001 (2)	0.024 (3)	−0.003 (2)
C18	0.025 (3)	0.024 (2)	0.031 (3)	0.000 (2)	0.008 (2)	0.003 (2)
C19	0.021 (3)	0.040 (3)	0.044 (3)	−0.002 (2)	0.017 (2)	0.004 (3)
C20	0.030 (3)	0.049 (4)	0.046 (4)	−0.003 (3)	0.024 (3)	−0.011 (3)
C21	0.036 (3)	0.031 (3)	0.046 (4)	0.003 (2)	0.023 (3)	−0.002 (3)
C22	0.027 (3)	0.025 (3)	0.026 (3)	0.000 (2)	0.009 (2)	−0.003 (2)
C23	0.031 (3)	0.019 (2)	0.018 (2)	0.006 (2)	0.010 (2)	0.000 (2)
C24	0.034 (3)	0.023 (2)	0.028 (3)	0.001 (2)	0.011 (2)	−0.002 (2)
C25	0.043 (3)	0.022 (3)	0.026 (3)	−0.004 (2)	0.009 (3)	−0.003 (2)
C26	0.035 (3)	0.024 (2)	0.021 (3)	0.000 (2)	0.008 (2)	0.005 (2)
C27	0.040 (3)	0.027 (3)	0.041 (3)	−0.007 (2)	0.015 (3)	0.002 (2)
C28	0.032 (3)	0.042 (3)	0.044 (4)	−0.006 (3)	0.018 (3)	0.006 (3)
C29	0.034 (3)	0.036 (3)	0.039 (3)	−0.003 (2)	0.018 (3)	0.006 (2)
C30	0.025 (3)	0.027 (3)	0.020 (3)	0.001 (2)	0.008 (2)	0.003 (2)
C31	0.028 (3)	0.035 (3)	0.026 (3)	0.001 (2)	0.005 (2)	0.005 (2)
C32	0.034 (3)	0.027 (3)	0.021 (3)	−0.002 (2)	0.007 (2)	0.000 (2)
C33	0.029 (3)	0.043 (3)	0.024 (3)	0.011 (2)	0.010 (2)	0.000 (2)
C34	0.026 (3)	0.071 (5)	0.027 (3)	0.020 (3)	0.013 (2)	0.007 (3)
C35	0.028 (3)	0.065 (5)	0.042 (4)	−0.001 (3)	0.023 (3)	0.015 (3)
C36	0.023 (3)	0.052 (4)	0.037 (3)	−0.007 (2)	0.010 (3)	0.010 (3)
C37	0.022 (2)	0.031 (3)	0.018 (2)	−0.001 (2)	0.005 (2)	0.003 (2)
C38	0.023 (3)	0.025 (2)	0.025 (3)	−0.003 (2)	0.007 (2)	−0.002 (2)
C39	0.038 (3)	0.029 (3)	0.036 (3)	0.002 (2)	0.009 (3)	0.002 (2)
C40	0.054 (4)	0.024 (3)	0.043 (4)	0.010 (3)	0.013 (3)	−0.002 (3)
C41	0.037 (3)	0.037 (3)	0.030 (3)	0.014 (3)	0.006 (3)	−0.005 (2)
C42	0.051 (4)	0.052 (4)	0.031 (3)	0.028 (3)	0.010 (3)	−0.004 (3)
C43	0.036 (4)	0.085 (6)	0.031 (4)	0.033 (4)	0.008 (3)	−0.006 (3)
C44	0.024 (3)	0.078 (5)	0.031 (3)	0.005 (3)	0.009 (3)	−0.013 (3)
C45	0.021 (2)	0.031 (3)	0.024 (3)	0.008 (2)	0.007 (2)	−0.004 (2)
C46	0.030 (3)	0.021 (2)	0.031 (3)	−0.004 (2)	0.011 (2)	−0.002 (2)
C47	0.031 (3)	0.041 (3)	0.023 (3)	0.001 (2)	0.006 (2)	0.004 (2)
C48	0.029 (3)	0.065 (4)	0.029 (3)	0.005 (3)	0.012 (3)	0.011 (3)
C49	0.030 (3)	0.100 (7)	0.029 (3)	0.009 (4)	0.017 (3)	0.013 (4)
C50	0.035 (4)	0.106 (7)	0.035 (4)	0.021 (4)	0.015 (3)	−0.009 (4)
C51	0.044 (4)	0.067 (5)	0.041 (4)	0.015 (3)	0.021 (3)	−0.009 (3)
C52	0.026 (3)	0.044 (3)	0.026 (3)	0.011 (2)	0.005 (2)	−0.008 (2)
C53	0.030 (3)	0.026 (3)	0.020 (3)	0.011 (2)	0.007 (2)	0.002 (2)
C54	0.045 (4)	0.028 (3)	0.025 (3)	0.014 (2)	0.002 (3)	−0.009 (2)
C55	0.035 (3)	0.024 (3)	0.049 (4)	−0.003 (2)	0.005 (3)	−0.006 (3)
C56	0.038 (3)	0.017 (2)	0.035 (3)	−0.006 (2)	0.004 (3)	0.002 (2)
C57	0.034 (4)	0.034 (3)	0.060 (4)	−0.007 (3)	0.006 (3)	0.020 (3)
C58	0.031 (3)	0.044 (4)	0.065 (5)	0.002 (3)	0.024 (3)	0.024 (3)
C59	0.030 (3)	0.041 (3)	0.046 (4)	0.003 (2)	0.020 (3)	0.012 (3)
C60	0.029 (3)	0.022 (2)	0.021 (3)	−0.005 (2)	0.004 (2)	0.000 (2)

### Geometric parameters (Å, °)

**Table:**

Ru1—Cl1	2.3933 (12)	C20—C21	1.392 (10)
Ru1—Cl2	2.3968 (13)	C21—C22	1.394 (10)
Ru1—N1	2.124 (5)	C22—C23	1.474 (9)
Ru1—N2	2.148 (4)	C23—C24	1.398 (7)
Ru1—C1	1.901 (7)	C24—C25	1.361 (10)
Ru1—C2	1.880 (6)	C25—C26	1.408 (10)
Ru2—Cl3	2.3883 (13)	C26—C27	1.401 (10)
Ru2—Cl4	2.3992 (13)	C26—C30	1.416 (8)
Ru2—N4	2.121 (5)	C27—C28	1.365 (11)
Ru2—N5	2.129 (4)	C28—C29	1.390 (9)
Ru2—C16	1.903 (7)	C33—C34	1.375 (11)
Ru2—C17	1.865 (6)	C34—C35	1.369 (11)
Ru3—Cl5	2.3996 (13)	C35—C36	1.389 (11)
Ru3—Cl6	2.3955 (12)	C36—C37	1.394 (10)
Ru3—N7	2.113 (5)	C37—C38	1.469 (9)
Ru3—N8	2.160 (4)	C38—C39	1.403 (8)
Ru3—C31	1.900 (7)	C39—C40	1.365 (11)
Ru3—C32	1.851 (6)	C40—C41	1.406 (11)
Ru4—Cl7	2.3955 (13)	C41—C42	1.416 (12)
Ru4—Cl8	2.3953 (13)	C41—C45	1.434 (9)
Ru4—N10	2.124 (5)	C42—C43	1.352 (12)
Ru4—N11	2.128 (4)	C43—C44	1.396 (12)
Ru4—C46	1.884 (7)	C48—C49	1.383 (12)
Ru4—C47	1.863 (6)	C49—C50	1.375 (14)
O1—C1	1.126 (9)	C50—C51	1.372 (13)
O2—C2	1.129 (7)	C51—C52	1.387 (11)
O3—C16	1.129 (9)	C52—C53	1.462 (10)
O4—C17	1.131 (7)	C53—C54	1.425 (8)
O5—C31	1.136 (9)	C54—C55	1.358 (11)
O6—C32	1.150 (7)	C55—C56	1.401 (10)
O7—C46	1.132 (9)	C56—C57	1.419 (11)
O8—C47	1.140 (8)	C56—C60	1.415 (8)
N1—C3	1.340 (9)	C57—C58	1.344 (11)
N1—C7	1.354 (7)	C58—C59	1.402 (9)
N2—C8	1.331 (8)	C3—H1	0.950
N2—C15	1.368 (8)	C4—H2	0.950
N3—C14	1.329 (9)	C5—H3	0.950
N3—C15	1.357 (8)	C6—H4	0.950
N4—C18	1.337 (8)	C9—H5	0.950
N4—C22	1.352 (7)	C10—H6	0.950
N5—C23	1.345 (8)	C12—H7	0.950
N5—C30	1.373 (8)	C13—H8	0.950
N6—C29	1.314 (9)	C14—H9	0.950
N6—C30	1.357 (8)	C18—H10	0.950
N7—C33	1.344 (9)	C19—H11	0.950
N7—C37	1.363 (7)	C20—H12	0.950
N8—C38	1.335 (8)	C21—H13	0.950
N8—C45	1.372 (8)	C24—H14	0.950
N9—C44	1.324 (10)	C25—H15	0.950
N9—C45	1.344 (9)	C27—H16	0.950
N10—C48	1.339 (10)	C28—H17	0.950
N10—C52	1.360 (8)	C29—H18	0.950
N11—C53	1.336 (8)	C33—H19	0.950
N11—C60	1.377 (9)	C34—H20	0.950
N12—C59	1.308 (10)	C35—H21	0.950
N12—C60	1.348 (8)	C36—H22	0.950
C3—C4	1.389 (10)	C39—H23	0.950
C4—C5	1.364 (9)	C40—H24	0.950
C5—C6	1.386 (10)	C42—H25	0.950
C6—C7	1.396 (9)	C43—H26	0.950
C7—C8	1.485 (9)	C44—H27	0.950
C8—C9	1.392 (8)	C48—H28	0.950
C9—C10	1.359 (10)	C49—H29	0.950
C10—C11	1.422 (9)	C50—H30	0.950
C11—C12	1.411 (10)	C51—H31	0.950
C11—C15	1.412 (8)	C54—H32	0.950
C12—C13	1.354 (10)	C55—H33	0.950
C13—C14	1.400 (9)	C57—H34	0.950
C18—C19	1.380 (10)	C58—H35	0.950
C19—C20	1.364 (9)	C59—H36	0.950
			
Cl1—Ru1—Cl2	174.66 (6)	C25—C26—C30	117.7 (6)
Cl1—Ru1—N1	87.63 (11)	C27—C26—C30	117.7 (6)
Cl1—Ru1—N2	91.66 (10)	C26—C27—C28	118.2 (5)
Cl1—Ru1—C1	89.55 (16)	C27—C28—C29	120.3 (7)
Cl1—Ru1—C2	92.51 (15)	N6—C29—C28	123.6 (7)
Cl2—Ru1—N1	87.24 (11)	N5—C30—N6	115.4 (5)
Cl2—Ru1—N2	85.67 (10)	N5—C30—C26	121.7 (6)
Cl2—Ru1—C1	95.47 (16)	N6—C30—C26	123.0 (6)
Cl2—Ru1—C2	89.65 (15)	Ru3—C31—O5	175.1 (6)
N1—Ru1—N2	76.2 (2)	Ru3—C32—O6	176.9 (5)
N1—Ru1—C1	175.1 (2)	N7—C33—C34	122.2 (6)
N1—Ru1—C2	97.7 (2)	C33—C34—C35	119.5 (7)
N2—Ru1—C1	99.9 (2)	C34—C35—C36	119.2 (7)
N2—Ru1—C2	172.5 (2)	C35—C36—C37	119.3 (6)
C1—Ru1—C2	86.4 (2)	N7—C37—C36	120.5 (6)
Cl3—Ru2—Cl4	174.31 (5)	N7—C37—C38	115.7 (5)
Cl3—Ru2—N4	88.64 (11)	C36—C37—C38	123.8 (5)
Cl3—Ru2—N5	86.68 (10)	N8—C38—C37	115.6 (5)
Cl3—Ru2—C16	88.23 (16)	N8—C38—C39	122.8 (6)
Cl3—Ru2—C17	92.39 (16)	C37—C38—C39	121.6 (6)
Cl4—Ru2—N4	87.73 (11)	C38—C39—C40	119.8 (6)
Cl4—Ru2—N5	88.21 (11)	C39—C40—C41	119.1 (6)
Cl4—Ru2—C16	95.10 (16)	C40—C41—C42	124.1 (6)
Cl4—Ru2—C17	92.31 (16)	C40—C41—C45	118.9 (7)
N4—Ru2—N5	76.7 (2)	C42—C41—C45	117.0 (6)
N4—Ru2—C16	175.2 (2)	C41—C42—C43	118.2 (7)
N4—Ru2—C17	95.3 (2)	C42—C43—C44	120.3 (8)
N5—Ru2—C16	99.5 (2)	N9—C44—C43	124.3 (7)
N5—Ru2—C17	171.9 (2)	N8—C45—N9	115.9 (5)
C16—Ru2—C17	88.6 (3)	N8—C45—C41	120.3 (6)
Cl5—Ru3—Cl6	175.22 (6)	N9—C45—C41	123.7 (6)
Cl5—Ru3—N7	87.61 (11)	Ru4—C46—O7	174.4 (5)
Cl5—Ru3—N8	93.47 (10)	Ru4—C47—O8	177.7 (7)
Cl5—Ru3—C31	89.95 (16)	N10—C48—C49	122.1 (7)
Cl5—Ru3—C32	90.91 (16)	C48—C49—C50	119.0 (8)
Cl6—Ru3—N7	87.61 (11)	C49—C50—C51	119.2 (8)
Cl6—Ru3—N8	85.42 (10)	C50—C51—C52	119.8 (7)
Cl6—Ru3—C31	94.82 (16)	N10—C52—C51	120.7 (6)
Cl6—Ru3—C32	89.63 (15)	N10—C52—C53	115.7 (6)
N7—Ru3—N8	76.2 (2)	C51—C52—C53	123.6 (6)
N7—Ru3—C31	175.4 (2)	N11—C53—C52	115.8 (5)
N7—Ru3—C32	96.8 (2)	N11—C53—C54	120.9 (6)
N8—Ru3—C31	100.1 (2)	C52—C53—C54	123.3 (6)
N8—Ru3—C32	171.6 (2)	C53—C54—C55	120.1 (6)
C31—Ru3—C32	87.1 (3)	C54—C55—C56	120.0 (5)
Cl7—Ru4—Cl8	174.97 (5)	C55—C56—C57	125.0 (5)
Cl7—Ru4—N10	89.32 (12)	C55—C56—C60	118.0 (6)
Cl7—Ru4—N11	89.08 (11)	C57—C56—C60	116.9 (6)
Cl7—Ru4—C46	91.14 (16)	C56—C57—C58	119.7 (6)
Cl7—Ru4—C47	92.41 (16)	C57—C58—C59	118.2 (7)
Cl8—Ru4—N10	88.85 (12)	N12—C59—C58	125.1 (7)
Cl8—Ru4—N11	85.94 (11)	N11—C60—N12	115.6 (5)
Cl8—Ru4—C46	90.35 (16)	N11—C60—C56	121.5 (6)
Cl8—Ru4—C47	92.45 (16)	N12—C60—C56	122.9 (6)
N10—Ru4—N11	76.5 (2)	N1—C3—H1	119.1
N10—Ru4—C46	175.9 (2)	C4—C3—H1	119.1
N10—Ru4—C47	96.6 (2)	C3—C4—H2	119.9
N11—Ru4—C46	99.4 (2)	C5—C4—H2	119.9
N11—Ru4—C47	172.9 (2)	C4—C5—H3	120.6
C46—Ru4—C47	87.5 (3)	C6—C5—H3	120.6
Ru1—N1—C3	125.2 (4)	C5—C6—H4	120.4
Ru1—N1—C7	116.0 (4)	C7—C6—H4	120.4
C3—N1—C7	118.7 (5)	C8—C9—H5	119.8
Ru1—N2—C8	115.8 (4)	C10—C9—H5	119.8
Ru1—N2—C15	125.5 (3)	C9—C10—H6	120.6
C8—N2—C15	118.2 (4)	C11—C10—H6	120.6
C14—N3—C15	116.9 (5)	C11—C12—H7	120.3
Ru2—N4—C18	125.8 (4)	C13—C12—H7	120.4
Ru2—N4—C22	115.9 (4)	C12—C13—H8	120.7
C18—N4—C22	118.3 (5)	C14—C13—H8	120.7
Ru2—N5—C23	115.6 (3)	N3—C14—H9	117.7
Ru2—N5—C30	125.6 (4)	C13—C14—H9	117.7
C23—N5—C30	118.7 (4)	N4—C18—H10	118.3
C29—N6—C30	117.3 (5)	C19—C18—H10	118.3
Ru3—N7—C33	124.9 (4)	C18—C19—H11	120.6
Ru3—N7—C37	115.9 (4)	C20—C19—H11	120.6
C33—N7—C37	119.1 (5)	C19—C20—H12	120.5
Ru3—N8—C38	115.4 (4)	C21—C20—H12	120.5
Ru3—N8—C45	125.1 (4)	C20—C21—H13	120.3
C38—N8—C45	119.1 (5)	C22—C21—H13	120.3
C44—N9—C45	116.3 (6)	C23—C24—H14	120.0
Ru4—N10—C48	125.7 (4)	C25—C24—H14	120.0
Ru4—N10—C52	115.4 (4)	C24—C25—H15	120.1
C48—N10—C52	118.9 (6)	C26—C25—H15	120.1
Ru4—N11—C53	116.1 (4)	C26—C27—H16	120.9
Ru4—N11—C60	124.4 (4)	C28—C27—H16	120.9
C53—N11—C60	119.4 (4)	C27—C28—H17	119.9
C59—N12—C60	117.0 (5)	C29—C28—H17	119.8
Ru1—C1—O1	173.4 (5)	N6—C29—H18	118.2
Ru1—C2—O2	176.3 (5)	C28—C29—H18	118.2
N1—C3—C4	121.7 (5)	N7—C33—H19	118.9
C3—C4—C5	120.1 (6)	C34—C33—H19	118.9
C4—C5—C6	118.7 (6)	C33—C34—H20	120.2
C5—C6—C7	119.1 (5)	C35—C34—H20	120.2
N1—C7—C6	121.5 (6)	C34—C35—H21	120.4
N1—C7—C8	115.2 (5)	C36—C35—H21	120.4
C6—C7—C8	123.3 (5)	C35—C36—H22	120.3
N2—C8—C7	115.5 (5)	C37—C36—H22	120.3
N2—C8—C9	122.8 (6)	C38—C39—H23	120.1
C7—C8—C9	121.7 (6)	C40—C39—H23	120.1
C8—C9—C10	120.4 (6)	C39—C40—H24	120.5
C9—C10—C11	118.9 (5)	C41—C40—H24	120.4
C10—C11—C12	124.3 (5)	C41—C42—H25	120.9
C10—C11—C15	117.5 (6)	C43—C42—H25	120.9
C12—C11—C15	118.2 (6)	C42—C43—H26	119.8
C11—C12—C13	119.3 (5)	C44—C43—H26	119.8
C12—C13—C14	118.6 (6)	N9—C44—H27	117.9
N3—C14—C13	124.6 (6)	C43—C44—H27	117.9
N2—C15—N3	115.4 (5)	N10—C48—H28	118.9
N2—C15—C11	122.3 (5)	C49—C48—H28	118.9
N3—C15—C11	122.3 (6)	C48—C49—H29	120.5
Ru2—C16—O3	175.1 (4)	C50—C49—H29	120.5
Ru2—C17—O4	177.0 (7)	C49—C50—H30	120.4
N4—C18—C19	123.4 (5)	C51—C50—H30	120.4
C18—C19—C20	118.8 (6)	C50—C51—H31	120.1
C19—C20—C21	119.0 (7)	C52—C51—H31	120.1
C20—C21—C22	119.4 (6)	C53—C54—H32	119.9
N4—C22—C21	121.1 (6)	C55—C54—H32	119.9
N4—C22—C23	115.3 (6)	C54—C55—H33	120.0
C21—C22—C23	123.5 (5)	C56—C55—H33	120.0
N5—C23—C22	115.7 (4)	C56—C57—H34	120.2
N5—C23—C24	122.0 (6)	C58—C57—H34	120.2
C22—C23—C24	122.2 (6)	C57—C58—H35	120.9
C23—C24—C25	120.0 (6)	C59—C58—H35	120.9
C24—C25—C26	119.9 (5)	N12—C59—H36	117.4
C25—C26—C27	124.6 (5)	C58—C59—H36	117.4
			
Cl1—Ru1—N1—C3	−81.7 (3)	C45—N8—C38—C37	−178.1 (4)
Cl1—Ru1—N1—C7	101.7 (3)	C45—N8—C38—C39	1.9 (7)
Cl1—Ru1—N2—C8	−97.1 (3)	C44—N9—C45—N8	179.7 (4)
Cl1—Ru1—N2—C15	91.4 (3)	C44—N9—C45—C41	2.7 (7)
Cl2—Ru1—N1—C3	99.8 (3)	C45—N9—C44—C43	0.2 (6)
Cl2—Ru1—N1—C7	−76.8 (3)	Ru4—N10—C48—C49	178.0 (4)
Cl2—Ru1—N2—C8	78.3 (3)	Ru4—N10—C52—C51	−174.5 (4)
Cl2—Ru1—N2—C15	−93.3 (3)	Ru4—N10—C52—C53	4.7 (5)
N1—Ru1—N2—C8	−10.0 (3)	C48—N10—C52—C51	3.5 (7)
N1—Ru1—N2—C15	178.5 (3)	C48—N10—C52—C53	−177.2 (4)
N2—Ru1—N1—C3	−174.0 (4)	C52—N10—C48—C49	0.2 (7)
N2—Ru1—N1—C7	9.4 (3)	Ru4—N11—C53—C52	−6.1 (5)
C2—Ru1—N1—C3	10.5 (4)	Ru4—N11—C53—C54	176.1 (3)
C2—Ru1—N1—C7	−166.1 (3)	Ru4—N11—C60—N12	5.2 (6)
C1—Ru1—N2—C8	173.1 (3)	Ru4—N11—C60—C56	−175.1 (3)
C1—Ru1—N2—C15	1.5 (4)	C53—N11—C60—N12	−177.0 (4)
Cl3—Ru2—N4—C18	−99.1 (3)	C53—N11—C60—C56	2.8 (7)
Cl3—Ru2—N4—C22	80.1 (3)	C60—N11—C53—C52	175.9 (4)
Cl3—Ru2—N5—C23	−81.4 (3)	C60—N11—C53—C54	−1.9 (7)
Cl3—Ru2—N5—C30	94.7 (3)	C59—N12—C60—N11	−179.3 (4)
Cl4—Ru2—N4—C18	85.3 (3)	C59—N12—C60—C56	0.9 (7)
Cl4—Ru2—N4—C22	−95.5 (3)	C60—N12—C59—C58	−2.2 (8)
Cl4—Ru2—N5—C23	96.0 (3)	N1—C3—C4—C5	0.6 (7)
Cl4—Ru2—N5—C30	−87.8 (3)	C3—C4—C5—C6	−1.6 (7)
N4—Ru2—N5—C23	7.9 (3)	C4—C5—C6—C7	0.0 (6)
N4—Ru2—N5—C30	−175.9 (4)	C5—C6—C7—N1	2.6 (7)
N5—Ru2—N4—C18	174.0 (4)	C5—C6—C7—C8	−176.2 (4)
N5—Ru2—N4—C22	−6.8 (3)	N1—C7—C8—N2	−0.9 (6)
C17—Ru2—N4—C18	−6.8 (4)	N1—C7—C8—C9	−178.6 (4)
C17—Ru2—N4—C22	172.3 (3)	C6—C7—C8—N2	177.9 (4)
C16—Ru2—N5—C23	−169.1 (3)	C6—C7—C8—C9	0.3 (6)
C16—Ru2—N5—C30	7.1 (4)	N2—C8—C9—C10	1.4 (7)
Cl5—Ru3—N7—C33	−78.0 (3)	C7—C8—C9—C10	178.9 (4)
Cl5—Ru3—N7—C37	103.2 (3)	C8—C9—C10—C11	−1.6 (7)
Cl5—Ru3—N8—C38	−96.3 (3)	C9—C10—C11—C12	179.7 (5)
Cl5—Ru3—N8—C45	91.0 (3)	C9—C10—C11—C15	1.4 (7)
Cl6—Ru3—N7—C33	101.9 (3)	C10—C11—C12—C13	179.9 (3)
Cl6—Ru3—N7—C37	−76.8 (3)	C10—C11—C15—N2	−1.2 (7)
Cl6—Ru3—N8—C38	79.1 (3)	C10—C11—C15—N3	179.2 (4)
Cl6—Ru3—N8—C45	−93.7 (3)	C12—C11—C15—N2	−179.5 (4)
N7—Ru3—N8—C38	−9.6 (3)	C12—C11—C15—N3	0.8 (7)
N7—Ru3—N8—C45	177.7 (3)	C15—C11—C12—C13	−1.8 (7)
N8—Ru3—N7—C33	−172.2 (4)	C11—C12—C13—C14	2.0 (7)
N8—Ru3—N7—C37	9.1 (3)	C12—C13—C14—N3	−1.2 (8)
C32—Ru3—N7—C33	12.6 (4)	N4—C18—C19—C20	−1.8 (8)
C32—Ru3—N7—C37	−166.2 (3)	C18—C19—C20—C21	1.0 (8)
C31—Ru3—N8—C38	173.2 (3)	C19—C20—C21—C22	0.8 (8)
C31—Ru3—N8—C45	0.4 (4)	C20—C21—C22—N4	−2.0 (7)
Cl7—Ru4—N10—C48	87.0 (4)	C20—C21—C22—C23	174.9 (4)
Cl7—Ru4—N10—C52	−95.1 (3)	N4—C22—C23—N5	2.0 (6)
Cl7—Ru4—N11—C53	96.0 (3)	N4—C22—C23—C24	178.4 (4)
Cl7—Ru4—N11—C60	−86.1 (3)	C21—C22—C23—N5	−175.0 (4)
Cl8—Ru4—N10—C48	−97.7 (4)	C21—C22—C23—C24	1.4 (7)
Cl8—Ru4—N10—C52	80.2 (3)	N5—C23—C24—C25	0.9 (7)
Cl8—Ru4—N11—C53	−83.3 (3)	C22—C23—C24—C25	−175.3 (4)
Cl8—Ru4—N11—C60	94.6 (3)	C23—C24—C25—C26	−1.6 (7)
N10—Ru4—N11—C53	6.5 (3)	C24—C25—C26—C27	−179.0 (5)
N10—Ru4—N11—C60	−175.6 (4)	C24—C25—C26—C30	2.2 (7)
N11—Ru4—N10—C48	176.2 (4)	C25—C26—C27—C28	−177.9 (5)
N11—Ru4—N10—C52	−5.9 (3)	C25—C26—C30—N5	−2.1 (7)
C47—Ru4—N10—C48	−5.4 (4)	C25—C26—C30—N6	177.8 (4)
C47—Ru4—N10—C52	172.5 (3)	C27—C26—C30—N5	179.0 (4)
C46—Ru4—N11—C53	−173.0 (3)	C27—C26—C30—N6	−1.1 (7)
C46—Ru4—N11—C60	4.9 (4)	C30—C26—C27—C28	0.9 (7)
Ru1—N1—C3—C4	−174.6 (3)	C26—C27—C28—C29	−0.3 (7)
Ru1—N1—C7—C6	173.3 (3)	C27—C28—C29—N6	−0.2 (7)
Ru1—N1—C7—C8	−7.7 (5)	N7—C33—C34—C35	−0.2 (6)
C3—N1—C7—C6	−3.5 (6)	C33—C34—C35—C36	−1.5 (8)
C3—N1—C7—C8	175.4 (4)	C34—C35—C36—C37	0.4 (8)
C7—N1—C3—C4	1.9 (7)	C35—C36—C37—N7	2.2 (7)
Ru1—N2—C8—C7	9.1 (5)	C35—C36—C37—C38	−175.0 (4)
Ru1—N2—C8—C9	−173.3 (3)	N7—C37—C38—N8	−0.9 (6)
Ru1—N2—C15—N3	−7.9 (5)	N7—C37—C38—C39	179.1 (4)
Ru1—N2—C15—C11	172.4 (3)	C36—C37—C38—N8	176.5 (4)
C8—N2—C15—N3	−179.3 (4)	C36—C37—C38—C39	−3.5 (7)
C8—N2—C15—C11	1.0 (6)	N8—C38—C39—C40	−2.1 (8)
C15—N2—C8—C7	−178.7 (4)	C37—C38—C39—C40	177.9 (5)
C15—N2—C8—C9	−1.1 (7)	C38—C39—C40—C41	1.6 (8)
C14—N3—C15—N2	−179.7 (4)	C39—C40—C41—C42	179.8 (5)
C14—N3—C15—C11	0.1 (5)	C39—C40—C41—C45	−1.0 (8)
C15—N3—C14—C13	0.2 (6)	C40—C41—C42—C43	179.6 (5)
Ru2—N4—C18—C19	179.8 (3)	C40—C41—C45—N8	0.8 (7)
Ru2—N4—C22—C21	−178.0 (3)	C40—C41—C45—N9	177.7 (5)
Ru2—N4—C22—C23	5.0 (5)	C42—C41—C45—N8	−179.9 (3)
C18—N4—C22—C21	1.3 (7)	C42—C41—C45—N9	−3.0 (7)
C18—N4—C22—C23	−175.8 (4)	C45—C41—C42—C43	0.4 (7)
C22—N4—C18—C19	0.6 (7)	C41—C42—C43—C44	2.3 (8)
Ru2—N5—C23—C22	−7.9 (5)	C42—C43—C44—N9	−2.8 (9)
Ru2—N5—C23—C24	175.6 (3)	N10—C48—C49—C50	−3.7 (8)
Ru2—N5—C30—N6	5.5 (5)	C48—C49—C50—C51	3.3 (9)
Ru2—N5—C30—C26	−174.6 (3)	C49—C50—C51—C52	0.3 (7)
C23—N5—C30—N6	−178.5 (4)	C50—C51—C52—N10	−3.8 (8)
C23—N5—C30—C26	1.4 (6)	C50—C51—C52—C53	177.0 (5)
C30—N5—C23—C22	175.7 (4)	N10—C52—C53—N11	0.9 (6)
C30—N5—C23—C24	−0.8 (6)	N10—C52—C53—C54	178.7 (4)
C29—N6—C30—N5	−179.5 (4)	C51—C52—C53—N11	−179.9 (3)
C29—N6—C30—C26	0.5 (7)	C51—C52—C53—C54	−2.1 (8)
C30—N6—C29—C28	0.1 (6)	N11—C53—C54—C55	0.2 (6)
Ru3—N7—C33—C34	−175.9 (3)	C52—C53—C54—C55	−177.4 (5)
Ru3—N7—C37—C36	175.0 (3)	C53—C54—C55—C56	0.7 (8)
Ru3—N7—C37—C38	−7.6 (5)	C54—C55—C56—C57	178.7 (5)
C33—N7—C37—C36	−3.9 (6)	C54—C55—C56—C60	0.1 (6)
C33—N7—C37—C38	173.6 (4)	C55—C56—C57—C58	−176.7 (6)
C37—N7—C33—C34	2.9 (7)	C55—C56—C60—N11	−1.9 (7)
Ru3—N8—C38—C37	8.7 (5)	C55—C56—C60—N12	177.9 (5)
Ru3—N8—C38—C39	−171.3 (3)	C57—C56—C60—N11	179.4 (5)
Ru3—N8—C45—N9	−5.9 (6)	C57—C56—C60—N12	−0.8 (8)
Ru3—N8—C45—C41	171.2 (3)	C60—C56—C57—C58	1.9 (8)
C38—N8—C45—N9	−178.3 (4)	C56—C57—C58—C59	−3.0 (9)
C38—N8—C45—C41	−1.2 (6)	C57—C58—C59—N12	3.3 (9)
